# Voice perturbations under the stress overload in young individuals: phenotyping and suboptimal health as predictors for cascading pathologies

**DOI:** 10.1007/s13167-020-00229-8

**Published:** 2020-11-12

**Authors:** A. Kunin, N. Sargheini, C. Birkenbihl, N. Moiseeva, Holger Fröhlich, Olga Golubnitschaja

**Affiliations:** 1 Departments of Maxillofacial Surgery and Hospital Dentistry, Voronezh N.N. Burdenko State Medical University, Voronezh, Russia; 2 Center of Molecular Biotechnology, CEMBIO, Friedrich-Wilhelms-Universität Bonn, Bonn, Germany; 3 Department of Bioinformatics, Fraunhofer Institute for Algorithms and Scientific Computing (SCAI), Schloss Birlinghoven, 53757 Sankt Augustin, Germany; 4 Bonn-Aachen International Center for IT, Rheinische Friedrich-Wilhelms-Universität Bonn, 53115 Bonn, Germany; 5 Predictive, Preventive and Personalised (3P) Medicine, Department of Radiation Oncology, University Hospital Bonn, Friedrich-Wilhelms-Universität Bonn, Bonn, Germany

**Keywords:** Predictive preventive personalised medicine, Voice perturbation, Suboptimal health, Stress, survey, Primary vascular dysregulation, Vasospasm, Biomarker pattern, Individualised patient profile, Phenotyping, Flammer syndrome, Body mass index, Underweight, Dry mouth syndrome, Hyposalivation, Xerostomia, Sicca syndrome, High altitude sickness, Tinnitus, Sense regulation, Pain sensitivity, Exercise-induced hypoalgesia, Microcirculation, Thirst, Circadian rhythm, Otorhinolaryngologoical disorders, Disease predisposition, Machine learning models, Association, Risk factors, COVID-19, Respiratory complications, Population screening, Pandemic, Risk assessment, Lifestyle intervention, Healthcare, Artificial intelligence (AI)

## Abstract

Verbal communication is one of the most sophisticated human motor skills reflecting both—the mental and physical health of an individual. Voice parameters and quality changes are usually secondary towards functional and/or structural laryngological alterations under specific systemic processes, syndrome and pathologies. These include but are not restricted to dry mouth and Sicca syndromes, body dehydration, hormonal alterations linked to pubertal, menopausal, and andropausal status, respiratory disorders, gastrointestinal reflux, autoimmune diseases, endocrinologic disorders, underweight versus overweight and obesity, and diabetes mellitus. On the other hand, it is well-established that stress overload is a significant risk factor of cascading pathologies, including but not restricted to neurodegenerative and psychiatric disorders, diabetes mellitus, cardiovascular disease, stroke, and cancers. Our current study revealed voice perturbations under the stress overload as a potentially useful biomarker to identify individuals in suboptimal health conditions who might be strongly predisposed to associated pathologies. Contextually, extended surveys applied in the population might be useful to identify, for example, persons at high risk for respiratory complications under pandemic conditions such as COVID-19. Symptoms of dry mouth syndrome, disturbed microcirculation, altered sense regulation, shifted circadian rhythm, and low BMI were positively associated with voice perturbations under the stress overload. Their functional interrelationships and relevance for cascading associated pathologies are presented in the article. Automated analysis of voice recordings via artificial intelligence (AI) has a potential to derive digital biomarkers. Further, predictive machine learning models should be developed that allows for detecting a suboptimal health condition based on voice recordings, ideally in an automated manner using derived digital biomarkers. Follow-up stratification and monitoring of individuals in suboptimal health conditions are recommended using disease-specific cell-free nucleic acids (ccfDNA, ctDNA, mtDNA, miRNA) combined with metabolic patterns detected in body fluids. Application of the cost-effective targeted prevention within the phase of reversible health damage is recommended based on the individualised patient profiling.

## Introduction

### Human voice reflects both—mental and physical health of individuals

Verbal communication is one of the most sophisticated human motor skills. Hundreds of muscles, innervated by a different network of the cranial and spinal nerve, subcortical, and cortical parts of the brain are involved in the production and processing of voice [1]. Contextually, the human voice is a complex phenomenon reflecting both—the mental and physical health of an individual [2]. According to the American Academy of Otolaryngology-Head and Neck Surgery Foundation, voice perturbations are defined as any abnormalities in the vocal quality, pitch, loudness, and vocal effort that influence communication or generate a negative effect on the voice-related quality of life [3, 4]. Table 1 exemplifies both—physiological and pathological factors associated with the voice perturbation.

**Table 1:** Physiological and pathological factors associated with the voice perturbation and disorders; information is modified from [5]

Factors	Prevalence	Literature source
Gender	• Higher prevalence of voice disorders in women	6
Ageing	• Increasing prevalence of laryngeal disorders in elderly (> 65 years old)	7
Puberty	• Prevalent alterations in voice parameters such as voice pitch	8
Family history of voice disorders	• Prevalent family history of hoarseness and voice disorders studied in certain professional groups such as teachers (*P* = 0.0001)	9
Professional occupation	• Voice perturbation and disorders are more prevalent in certain professional groups such as teachers, call centre workers and singers	10
Toxic environment	• Higher risk of voice perturbation and laryngeal disorder in (both active and passive) tobacco smokers	11, 12

Voice disturbances impact one in thirteen adults annually. According to the US National Ambulatory Medical Care Survey, almost 52% of adults revealed a voice problem that lasted more than a week [6]. Voice disturbances are among the most critical job-related hazards resulting from numerous vocal cord traumas among people who use their voice professionally, like teachers, singers, actors, telemarketers, salespersons, and nurses [7]. Among teachers in different parts of the world, the prevalence of voice disorders ranges from 20 to 80%, and the most usual symptoms include dry throat, hoarseness, and throat clearing [8]. A high number of individuals with vocal perturbations do not consult a physician because their experienced symptoms are impermanent, and the condition is considered unimportant [9]. Consequently, early disease stages remain clinically undetected.

### Health conditions and disorders with the laryngeal manifestation

Voice parameters and quality changes are usually secondary towards functional and/or structural laryngological alterations under certain systemic processes, syndrome, and pathologies. These include but are not restricted to Sicca syndrome [10], dry mouth syndrome [11] and body dehydration [12], hormonal alterations linked to pubertal [13], menopausal [14] and andropausal [15] status and acromegaly [16], cardio-vocal syndrome [17], respiratory disorders (e.g. chronic obstructive pulmonary disease [18] and asthma [19]), gastrointestinal reflux [20], autoimmune diseases such as rheumatic arthritis [21] and Sjögren’s syndrome [22], systemic lupus erythematosus [23], amyloidosis [24], cystic fibrosis [25], endocrinologic disorders such thyroid dysfunction [26, 27] and anorexia nervosa [28–30], underweight versus overweight and obesity [31, 32], and diabetes [33]. Table 2 highlights corresponding laryngeal symptoms.

**Table 2:** Laryngeal manifestations of systemic processes and pathologies; “+“means increased

Diseases	Laryngopharyngeal reflux diseases	Autoimmune diseases	Respiratory diseases (e.g. asthma bronchial)	Endocrine disorders (e.g. anorexia nervosa, hypothyroid)	BMI (overweight and obesity)	Neurological diseases	Systemic dehydration
Laryngeal manifestations							
Dry mouth	Unknown	+	+	+	+	+	+
MPT	Short	Short	Short	Short	Short	Short	Short
Shimmer	+	+	+	+	+	+	+
Jitter	+	+	+	+	+	+	+
Hoarseness	+	+	+	+	+	+	+
Roughness	+	+	+	+	+	+	+

### Voice as an indicator of the stress overload

It is well-established that stress overload or imbalanced stress (i.e. highly increased stress load combined with insufficient repair capacity) is a significant risk factor of cascading pathologies, including but not restricted to neurodegenerative and psychiatric disorders, cancer, diabetes mellitus, cardiovascular disease, and stroke [34]. Unfortunately, there is an evident lack of reliable measurables and standardised approaches to estimate stress overload at the individual level. Indeed, a survey performed amongst college students revealed vocal fatigue significantly increased for individuals suffering from psychological stress [35]. Furthermore, anxiety has been demonstrated as affecting acoustic parameters of voice [36], and psychological stress is clearly associated with vocal symptoms amongst professors who reported elevated stress that they experienced during previously given lessons [37].

### Primary vascular dysregulation/vasospasm as the stress facilitator

Vasospasm frequently caused by primary vascular dysregulation occurring even in adolescents and young adults carries a systemic character and plays a pivotal role in oxidative stress and cascading pathologies [38]. There are multifaceted risk factors including but not restricted to genetic predisposition and specific phenotypes such as Flammer syndrome (FS), which synergistically lead to the imbalance between strong vasoconstriction and insufficient vasodilation. Vasospastic reaction is particularly pronounced under specific stimuli such as the cold provocation, hormonal, and emotional stress [39]. Consequently, developed vasoconstriction carries a systemic character affecting both (cold) extremities and poor microcirculation in the life-important organs [40]. In turn, prolonged and frequent systemic hypoxia and ischemic lesions may lead to

- Endothelial dysfunction and cardiovascular disease CVD [41]
- Mitochondrial dysfunction [42]
- Chronic inflammation [43]
- Impaired healing [44]
- Autoimmune diseases [45]
- Stroke and other neurological disorders [46]
- Aggressive cancer subtypes and metastatic disease [47–49], amongst others.

### A link between vasospastic reaction and dry mouth syndrome

The coincidence and a potential functional link between vasospastic reaction and dry mouth syndrome have recently been detailed for Sjögren syndrome with cascading pathologies such as chronic inflammation and cancer predisposition [45]. Follow-up studies performed on young and healthy individuals revealed a statistically significant positive association between dry mouth syndrome/hyposalivation and several symptoms characteristic for the FS phenotype, such as decreased feeling of thirst but strongly pronounced reaction towards stress and imbalanced vasoconstriction [11].

## Working hypothesis

In the current study, we hypothesised that the voice perturbation under stress conditions might be positively associated with signs and symptoms of dry mouth syndrome and vascular dysregulation in otherwise healthy individuals. To verify the hypothesis, young individuals recruited for the study underwent a comprehensive survey based on questions relevant to dysregulation, phenotyping, dry mouth syndrome, and voice problems observed specifically under the stress overload.

## Materials and methods

### Study design

Altogether 200 students, 18–23 years of age, were recruited for this international study at the Voronezh N.N. Burdenko State Medical University, Voronezh, Russia. Gender status was not considered as stratification criteria in the design of the current study. Recruited participants were informed about the study’s purposes, have signed corresponding consent, and filled in the questionnaire.

### Dry mouth syndrome/hyposalivation

Dry mouth syndrome/hyposalivation was determined by the Bother xerostomia Index (BI) utilising a questionnaire of 10 issue-specific items [50].

### FS phenotype

Vasospasm relevant FS phenotype was characterised earlier [51]. The FS related items applied to the actual study have been developed at the University Hospital Basel, Switzerland [52].

### Comprehensive survey: phenotyping, symptoms, and signs of disturbed microcirculation and dry mouth syndrome relevant for suboptimal health and associated pathologies

Items used in the actual study utilised knowledge collected towards their functional interconnections as demonstrated in a series of previous publications:

- Individualised patient profiles—phenotyping and genotyping in the transitional phase between suboptimal health and disease [47, 53]
- Dry mouth syndrome in young individuals - potentially cascading pathologies [11]
- Vaginal dryness and potential diseases [54]
- Sjögren and Sicca syndrome [45]
- Phenotyping to assess risks of “young” stroke [46]
- Phenotyping to assess risks of cancer and metastatic disease development [47–49].

### Statistical analysis

To identify statistically significant associations between individual items used for the survey and the occurrence of voice problems, logistic regression was performed with objects representing the independent- and voice problems the dependent variables. The analysed data consisted of 200 participants, of which 45 (22.5%) reported voice problems. By applying a Wald-Test, we determined significant associations to a confidence level of 95% (i.e. *P* values smaller than 0.05 were considered significant). Multiple testing correction was performed via the Benjamini and Hochberg method. Participant’s age and gender were considered as covariates in the model such that statistical results are corrected for their potential influence on the outcome.

## Results

Statistical evaluation of the survey revealed 45 individuals (out of altogether 200 study participants) presenting 22.5% of the entire study population, who reported voice perturbations under stress conditions. Table 3 summarises statistically relevant symptoms associated with the occurrence of vocal problems under stress conditions. The signs of reported regression coefficients indicate a positive or negative association. Corresponding items have been grouped into five categories, namely

1. Signs and symptoms of dry mouth syndrome
2. Signs and symptoms of disturbed microcirculation
3. Altered sense regulation and shifted circadian rhythm
4. body shape
5. Prevalence of acute and chronic otorhinolaryngological disorders.

**Table 3:** Comparative presentation of factors and symptoms identified in the study population as being positively (positive regression coefficient) or negatively (negative regression coefficient − 3.7, otorhinolaryngological disorders) associated with voice perturbations under stress conditions; standard error and statistical significance (*P* value) are noted for each item; *P* ≤ 0.05 is considered significant

Items	Regression coefficient (standard error)	*P* value
Dry mouth syndrome		
Feeling of dry mouth at night or getting up	2.1 (0.9)	< 0.05
Problems with food tasting	7.8 (3.0)	< 0.01
Disturbed microcirculation		
High altitude sickness	2.2 (0.8)	< 0.01
Tinnitus	3.0 (0.8)	< 0.001
Altered sense regulation and shifted circadian rhythm		
Usually do not feel thirsty	1.9 (0.9)	< 0.05
Increased pain sensitivity	3.3 (0.9)	< 0.001
Circadian rhythm is shifted towards later hours in the night	2.5 (0.8)	< 0.01
Body shape		
BMI < 20 kg/m^2^	2.0 (0.8)	< 0.05
Prevalence of acute and chronic otorhinolaryngological disorders		
Frequent season and chronic infections	− 3.7 (1.2)	< 0.01

Thereby, categories 3 and 4 collectively describe the phenotype, which was found to be characteristic for individuals with the voice perturbation under stress conditions, namely low BMI, altered sense regulation (including the reduced feeling of thirst), and shifted circadian rhythm.

From the entire spectrum of dry mouth symptoms and signs described in the literature [50], only two were positively associated with the target group. However, reduced feeling of thirst may potentially cascade body dehydration leading to dry mouth and Sicca syndromes as detailed earlier [11, 54].

The relevance of the disturbed microcirculation was strongly supported by the positive association of both—high altitude sickness and tinnitus demonstrated for the target group with high level of statistical significance.

Noteworthy, the prevalence of acute and chronic otorhinolaryngological disorders was negatively associated with the target group that demonstrates otorhinolaryngological disorders as irrelevant for the voice perturbation under stress conditions in the study population.

### Case reports

#### Case 1

Nineteen-year-old female studying medicine, generally healthy; evident voice perturbation under the stress overload; particularly noticeable responses have been given towards following symptoms:

- Do not feel thirsty but dry mouth at night, waking up in the morning and during the day
- Evidently disturbed microcirculation (frequently observed cold extremities and dizziness)
- Circadian rhythms is shifted towards later hours in the night
- Sleep duration below 7 hours
- Evident skin blotches in stress situations

There are stroke cases and cases with aggressive metastatic cancers amongst relatives in the family.

#### Case 2

Twenty-one-year-old male studying medicine, generally healthy; evident voice perturbation under the stress overload; particularly noticeable responses have been given towards following symptoms:

- Strongly reduced feeling of thirst
- Dizziness
- Frequent migraine with accompanying symptoms such as migraine with aura
- Tinnitus
- Evident skin blotches in stress situations
- Sleep duration below 7 hours

There are stroke cases amongst relatives in the family.

#### Case 3

Eighteen-year-old female studying medicine, generally healthy; evident voice perturbation under the stress overload; particularly noticeable responses have been given towards following symptoms:

- Strongly reduced feeling of thirst
- Dizziness
- Frequent headache
- Strongly pronounced pain sensitivity
- Tinnitus
- Evident skin blotches in stress situations
- Circadian rhythms is shifted towards later hours in the night
- Sleep duration below 7 hours
- Sicca syndrome
- Wound healing is impaired

There are stroke cases amongst relatives in the family.

#### Case 4

Twenty-year-old female studying medicine, generally healthy; evident voice perturbation under the stress overload; particularly noticeable responses have been given towards following symptoms:

- BMI < 20 kg/m^2^
- Do not feel thirsty but dry mouth at night and waking up in the morning
- Evidently disturbed microcirculation (frequently observed cold extremities, dizziness, high altitude sickness)
- Frequent headache
- Tinnitus
- Circadian rhythms is shifted towards later hours in the night
- Sleep duration below 7 hours
- Wound healing is impaired

There are stroke cases amongst relatives in the family.

## Data interpretation

### Imbalanced stress conditions: the mechanism of cascading pathologies

Excessive release of reactive oxygen species (ROS) and reactive nitrogen species (RNS) have been identified as initiators, mediators, and regulators of cellular oxidative stress (OxiS). OxiS can damage almost any biomolecule, including chromosomal and mitochondrial DNA [55, 56]. Oxidative damage plays a vital role in cascading pathologies, which include but are not restricted to CVD [57], neurodegeneration [58–60], stroke [46], diabetes mellitus with a broad spectrum of complications [61], and cancers [43, 62] such as castration-resistant prostate cancer (via the androgen receptor (AR) dependent pathway) [63] and triple-negative breast cancer with aggressive metastatic diseases and particularly poor outcomes [47, 49]. To this end, both ROS and RNS can be carcinogenic by modifying the inflammatory status [43], influencing cellular lipid structures, angiogenesis, and antiapoptotic pathways, amongst others [62, 64].

### Stress overload negatively impacts microcirculation, hormonal profile, immune system, and mitochondrial function

#### Stress overload and disturbed microcirculation—the vicious circle by reciprocity

Any kind of stress, such as hormonal, psychological, and emotional, is known to cause vasoconstriction and disturbed microcirculation [65] leading, in turn, to systemic hypoxic-ischemic effects and extended oxidative stress [44, 66–68]. The reciprocity may result in a vicious circle increasing the extent of both—disturbed microcirculation and stress overload [69], which synergistically cascade a number of related pathologies [46, 47, 49, 63, 67, 70–72].

#### Stress overload and disturbed microcirculation synergistically lead to mitochondrial dysfunction and uncontrolled increase in ROS production and suppressed energy supply

Mitochondrial dysfunction can be caused by an extensive production of ROS resulting from synergistic effects of the stress overload and disturbed microcirculation [73] that is characteristic for ageing and related pathologies [74]. In turn, mitochondrial dysfunction leads to an uncontrolled increase in the ROS production and suppressed energy supply—both resulting in extended damage to life-important biomolecules such as chromosomal and mitochondrial DNA and diminished repair capacity as detailed in the literature [47]. Depending on the individual genetic setup and environmental risk factors, affected individuals are strongly predisposed to neurodegenerative pathologies such as Alzheimer’s disease or cancers [47, 75].

#### Stress overload, disturbed microcirculation, and accelerated ageing

Individuals extensively exposed to imbalanced stress are strongly predisposed to accelerated ageing—corresponding mechanisms have been described [76–78]. Consequently, affected systems are hormonal regulation and immune system—both involved in longevity versus ageing with cascading related pathologies [79–81].

#### Stress overload, disturbed microcirculation, and immune dysfunction

Stress-induced immune dysfunction [82] is well described in the literature. Chronic inflammation is a significant characteristic of the stress overload and disturbed microcirculation [44]. Potential implication is relevant for inflamm-ageing, acute infectious diseases [83, 84], chronic impairments [45], healing processes [44], and immune dysregulation in cancers [85], amongst others.

### High altitude sickness and tinnitus are attributable to disturbed microcirculation

#### High altitude sickness

Individuals with disturbed microcirculation demonstrate a prolonged adaptation to the changing altitude and a tendency towards high altitude sickness [86]. To this end, increased baroreceptor sensitivity has been demonstrated in the context of compromised cerebral blood flow and predisposition to the ischemic stroke [46, 87].

#### Tinnitus

Stress overload and vascular dysregulation of different origin plays the key role in pathogenesis of tinnitus [88]. Although corresponding pathomechanisms are still under-investigated, the evident relationship between vascular dysregulation and tinnitus is the reduced bioavailability of nitric oxide (NO) in vasospastic individuals. NO is reduced in the jugular vein of individuals with tinnitus, resulting in disruption of microcirculation in the ear [89].

### Pain sensitivity is related to the quality of microcirculation

Although corresponding regulatory mechanisms are not yet detailed in the literature, acupuncture’s effects demonstrate that pain sensation is reduced by improving microcirculation [90]. Further, regular physical exercises are useful for improving microcirculation used, for example, for early lifestyle interventions in children for primary prevention [91]. On the other hand, exercise-induced hypoalgesia (EIH) is a well-established phenomenon, specifically in pain-free individuals. EIH describes a decrease in pain sensitivity after an acute bout of exercise. EIH, as a pain modulation tool, has important implications in several medical areas [92, 93].

### Low BMI is relevant for cascading pathologies

Although physiologic body mass index (BMI) is a highly individual parameter, there are clear indications provided in the literature that BMI below 24 kg/m^2^ is relevant for an increased overall and cause-specific mortality as demonstrated, for example, by a population-based cohort study of 3.6 million adults in the UK [94]. A study focused on prostate cancer specific mortality and overall mortality concerning BMI demonstrated a statistically significant increased risk of prostate cancer specific mortality and overall mortality in the group with high BMI (≥ 27.5 kg/m^2^) as well as in the group with low BMI (< 22.5 kg/m^2^) compared to the reference group (BMI 22.5–25 kg/m^2^) [95]. Data collected from 22 clinical trials showed that BMI ≥ 25 kg/m^2^ was associated with better overall survival amongst prostate cancer patients compared to those with BMI < 25 kg/m^2^ [96]. Further, clear indication has been provided, demonstrating that underweight women (BMI < 20) are at a sufficiently higher risk for BC diagnosis and mortality compared to the standard range BMI = 20–25 [97].

### Dry mouth syndrome and body dehydration are relevant for both—voice changes and associated pathologies

Dry mouth syndrome can result from some pathophysiological conditions, like exposure to acute and chronic stress, eating disorders (such as anorexia nervosa), metabolic syndrome(s), Sjögren’s and Sicca syndromes, and head/neck radiotherapy application. In turn, hyposalivation may predispose individuals to chronic oral pain, dental caries, taste changes, halitosis, voice and digestive disorders, and burning mouth syndrome [11, 98]. Xerostomia may impact tissue viscosity of the vocal tract and oral mucosa and subsequently influence vocal function [12]. Roh et al. [99] demonstrated that voice range profiles of pitch and loudness reduced remarkably in individuals with xerostomia. Individuals with primary Sjögren’s syndrome and dry mouth show significantly increased voice handicap index [10]. Systemic dehydration and surface dehydration on vocal folds surface have a detrimental impact on tissue viscosity and mucosal wave and contribute to vocal fatigue [100, 101].

### 3PM-related conclusions and expert recommendations

The current study followed 3PM-related guidelines recommended by the European Association for Predictive, Preventive and Personalised Medicine [102]. In detail, the following 3PM strategies are recommended:

A. Voice perturbation under the stress overload is considered a potent biomarker for suboptimal health conditions being positively associated with symptoms of dry mouth syndrome, disturbed microcirculation, altered sense regulation, shifted circadian rhythm as well as BMI < 20 kg/m^2^. According to the currently available literature cited above, there are clear functional interrelationships, which, therefore, considered as being evidence-based. Relevant cascading pathologies include but are not restricted to Sicca syndrome and body dehydration, cardio-vascular and hormonal alterations, respiratory disorders, endocrinologic and autoimmune disorders, stroke, and cancers. Therefore, extended surveys applied in the population might be useful, for example, to identify persons at high risk for respiratory complications under pandemic conditions such as COVID-19.
B. In our study, voice was analysed via a classical survey-based approach. Automated analysis of voice recordings via artificial intelligence (AI) has a potential to derive digital biomarkers, which are less subjective than questionnaires and can be assessed also in an outpatient situation (e.g. via telephone) [103]. Further, predictive machine learning models should be developed that allows for detecting a suboptimal health condition based on voice recordings, ideally in an automated manner using digital biomarkers.
C. Follow-up stratification and monitoring of individuals in suboptimal health conditions identified by specialised surveys under point A and/or by AI under point B are recommended utilising disease specific cell-free nucleic acids (cfDNA, ctDNA, mtDNA, miRNA) combined with metabolic patterns detected in body fluids [43, 104–106]. To this end, severely affected cells and tissues intrinsically secrete cell-free nucleic acids [106] such as mitochondrial DNA fragments [107]. It has been demonstrated that COVID-19 patients with increased levels of mtDNA are at elevated death risk, necessity of ICU care and intubation [107].
D. Application of the cost-effective targeted prevention within the phase of reversible health damage is recommended based on the individualised patient profile created and risk assessment recommended under points A, B, and D. The most prominent examples for the targeted prevention against cancer development and progression have been recently published [56, 62, 64].

## Abbreviations

AI: artificial intelligence
AR: androgen receptor
BMI: body mass index
ccfDNA: circulating cell-free DNA
ctDNA: circulating tumour DNA
CVD: cardiovascular disease
EIH: exercise-induced hypoalgesia
FS: Flammer syndrome
MPT: maximum phonation time
miRNA: microRNA
mtDNA: mitochondrial DNA
NO: nitric oxide
OxiS: oxidative stress
PPPM/3PM: predictive preventive personalised medicine
RNS: reactive nitrogen species
ROS: reactive oxygen species
